# Trends in specialized palliative care referrals at an oncology center from 2007 to 2019

**DOI:** 10.1186/s12904-021-00828-w

**Published:** 2021-09-03

**Authors:** Lucka Boltezar, Barbara Jezersek Novakovic, Maja Ebert Moltara

**Affiliations:** 1 Department of Medical Oncology, Institute of Oncology Ljubljana, Zaloska cesta 2, Ljubljana, Slovenia; 2 Faculty of Medicine, University of Ljubljana, Vrazov trg 2, Ljubljana, Slovenia; 3 Acute Palliative Care Department, Institute of Oncology Ljubljana, Zaloska cesta 2, Ljubljana, Slovenia

**Keywords:** Palliative care, Referral-to-death interval, Primary site of cancer, Early referral

## Abstract

**Background:**

Early referral to palliative care, at least 3 months before death, should be a standard of care in oncological practice. Real life data in this setting are invaluable since they provide a picture of everyday practice and serve as the basis for future improvements.

**Methods:**

We conducted a retrospective cohort assessment of all patients referred to our specialized palliative care (SPC) services at the Institute of Oncology, Ljubljana, Slovenia. Our analysis includes patient referrals between 2007 and 2019.

**Results:**

During the above-specified time period of 13 years, 3234 patients were referred for SPC services at our institution. The median age at SPC referral was 67 years. The majority of patients (63%) were assessed only once, while 31% of patients were seen on more than one occasion. Median time from SPC referral to death was 25 days for the whole group. 1693 patients (52.7%) were referred to SPC in the last 30 days before death, 785 (25.8%) patients between 31 and 90 days and 652 (21.4%) patients more than 3 months before death. Neither age nor sex correlated with the duration of referral time. However, there was a strong correlation between the year of referral to palliative care and the duration of palliative care service (ρ = 0.19, *p* < 0.001). The median referral to death interval for lymphoma patients and breast cancer patients were 15 and 18 days, respectively, and the median referral to death interval for colorectal cancer and lung tumor patients were 34 and 26 days before death, respectively.

**Conclusion:**

Throughout the existence of our SPC services we have observed a positive trend in the number of referrals, a lengthening of time between referral and death, as well as an increase in the proportion of patients with an early referral to SPC (more than 3 months before death). Neither age nor sex correlated with the length of referral time.

## Background

Early integration of palliative care into standard oncological treatment of advanced cancer has been gaining in importance over the past few decades [1, 2]. Temel et al. were the first to emphasize early integration of palliative care into standard oncological care, leading to a better quality of life and better survival of patients with metastatic disease [3]. Early palliative care referral is defined as a referral 3 months or more before death and is associated with improved end-of-life care, fewer emergency room visits, hospitalisations and hospital deaths [4], as well as less aggressive procedures near the end of life [5, 6]. Longer duration of palliative care services has already been correlated with a longer survival time [1, 7]. The majority of real life studies [4–10] have highlighted local practices, underlying good practices as well as pointing to conditions in need of improvements. These studies mostly included a few hundred patients, with the exception of Bennett’s study [9] of 3900 cancer patients and a large cohort of patients with lung cancer analysed by Goldwasser [6]. Real life analyses of larger groups of cancer patients are necessary as they give a comprehensive overview of local practices.

The Institute of Oncology Ljubljana (IOL) is the major comprehensive cancer centre in Slovenia and performs the majority of oncological treatments for the population of Slovenia (2 million inhabitants). The official data for 2020 are: 15,300 admissions, 10,600 outpatient services and 11,700 newly referred patients [11]. Specialized palliative care (SPC) services at our institute have evolved gradually: first with the establishment of the Department of Acute Palliative Care (APCD) in 2007 and later with the introduction of an outpatient clinic and inpatient consultations in 2013. Our Department comprises seven in-patient beds, while mobile palliative care services are planned for the future. The palliative service at IOL only deals with cancer patients.

In order to obtain an overview of palliative care services from 2007 to 2019, we performed a retrospective cohort assessment of all patients referred for SPC services, including basic patient characteristics (age, sex, primary site of cancer), times of referrals and trends in referrals through time.

## Methods

We conducted a retrospective cohort assessment of all patients referred to our SPC services (inpatient, outpatient, inpatient consultations) at IOL between 2007 and 2019. All procedures followed in this assessment were in accordance with the ethical standards of the committee responsible for human experimentation (Ethics Committee of the Institute of Oncology Ljubljana, approval number: ERID-KSOPKR-0086/2020) and the Helsinki Declaration of 1975, as revised in 2000. The Ethics Committee of the Institute of Oncology Ljubljana waived the need for consent as this was a retrospective database analysis.

Data were gathered regarding sex, age at SPC referral, primary site of cancer (according to International Classification of diseases – ICD-10), date of first scheduled appointment at the SPC service (preceeded by a written refferal by the patient’s oncologist) and date of death. The category of services of palliative care (inpatient, outpatient, inpatient consultation) was recorded. In an outpatient setting, we also recorded missed appointments. Since the presence of the patient is never in question for inpatient and inpatient consultation SPC services, missed appointments were observed only in the outpatient setting. For each patient, the total number of palliative care services needed and all scheduled appointments were determined, taking into account any missed appointments. Individual patient's referral-to-death interval was calculated from the date of the first scheduled appointment at the SPC service by the patient’s oncologist (medical oncologist, surgical oncologist or radiation oncologist) to the date of death. The first scheduled appointment was chosen to be the starting point because of irregularities and difficulties in tracking all the dates of written referrals (a lot of written referrals lacked dates), there were also quite a few referrals by personal communication at the beginning of our practice. Referral-to-death intervals were subdivided into three groups: less than 30 days before death (very late), 31–90 days before death (late) and more than 90 days before death (early).

The dates of death were provided by the Cancer Registry of the Republic of Slovenia and survival status was assessed on July 15, 2020.

### Statistics

Descriptive statistics were used for baseline demographic data (median, standard deviation, percentage). Sex and age groups were analysed using chi-square and t-tests. For continuous, nonparametric variables, the Mann–Whitney test and Kruskal Wallis tests were applied. Correlation coefficients of age, sex, cancer diagnosis, year of referral to palliative care, and total duration of palliative care service were analyzed by Spearman’s correlation analyses. Statistical analyses were performed using IBM SPSS Statistics, version 26. A two-sided *p* value of 0.05 was considered significant.

## Results

### Patient characteristics

The cohort consisted of 3234 patients who were engaged in our SPC services between 2007 and 2019. There was a minor male predominance with 1656 (51.2%) males and 1578 (48.8%) females included in the services. At the time of data collection, 97.6% of patients were deceased, while 72 (2.2%) patients were alive. Five patients were lost to follow-up in the Cancer Registry of Republic of Slovenia.

Median age at the time of referral to SPC services was 67 years old, range 21 – 95, with no difference between males and females.

The distribution according to patient age is presented in Table 1.

**Table 1:** Distribution of patients according to age at time of SPC referral

Age group	Number of patients (%)
15–40	121 (3.7)
41–65	1327 (41.0)
66–75	968 (29.9)
76–85	701 (21.7)
86–100	117 (3.6)

The frequency of different primary sites of cancer is given in Table 2.

**Table 2:** Distribution of patients by cancer diagnosis, according to the International Classification of Diseases – ICD-10

Primary site of neoplasm (ICD-10 code)	Number of patients (%)
Lip, oral cavity and pharynx (C0-C14)	110 (3.4)
Oesophagus (C15)	62 (1.9)
Stomach (C16)	177 (5.5)
Small intestine (C17)	13 (0.4)
Colorectal (C18-C20)	506 (15.6)
Anus and anal canal (C21)	8 (0.2)
Hepatobilliary tract (C22-C24)	30 (0.9)
Pancreas (C25)	107 (3.3)
Digestive organs, other (C26)	1 (0.03)
Upper respiratory tract (C30-C33)	16 (0.5)
Bronchus and lung (C34)	523 (16.2)
Intrathoracic organs, other (C37-C39)	6 (0.2)
Bone and articular cartilage (C40-C41)	14 (0.4)
Skin, including melanoma (C43-C44)	193 (6.0)
Mesothelial and soft tissue (C45-C49)	84 (2.6)
Breast (C50)	324 (10.0)
Female genital organs (C51-C58)	275 (8.5)
Male genital organs (C60-C63)	208 (6.4)
Urinary tract (C64-C68)	237 (7.3)
Eye, brain and other parts of central nervous system (C69-C72)	59 (1.8)
Thyroid and other endocrine glands (C73-C75)	20 (0.6)
Cancer of unknown origin (C76-C80)	94 (2.9)
Hematologic (C81-C96)	167 (5.2)

**Fig. 1:**
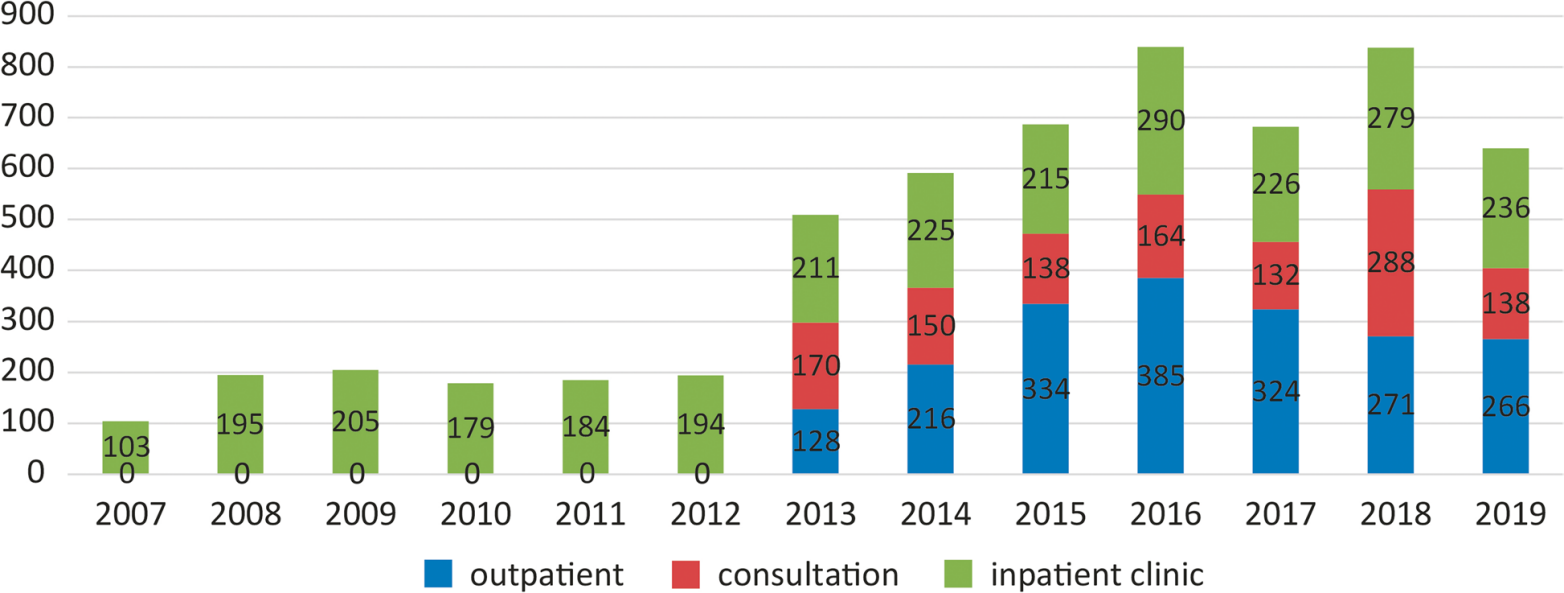
Number of annual services (outpatient, inpatient and inpatient consultations)

SPC services were provided 5851 times, ranging from 1 to 22 visits/consultations per patient. The number of annual services is presented in Fig. 1.

About two-thirds of patients (62.7%) had one visit, 558 (17.3%) patients two visits, 205 (6.3%) patients three visits and 793 (7.3%) patients four or more visits with the SPC team. Two hundred and eight patients (6.4%) were scheduled for outpatient visits, yet underwent none – they did not attend the scheduled meetings on one or more occasions. Almost half of the performed services of the SPC team were inpatient at the APCD (46.8%), 33.0% were outpatient, while 20.2% were inpatient consultations.

### Timing of specialised palliative care referral (referral-to-death interval)

The median referral-to-death interval was 25 days for the whole group, range minus 110 to 1831 days.

There were some “negative time” referrals in 117 patients (3.7%), representing those scheduled for an outpatient visit after their death. Fifty two patients (1.6%) were presented to the SPC team for the first time on the day of their death and 2988 patients (94.6%) were referred to SPC one or more days before death.

Fifty two point seven percent of patients (*N* = 1603) were referred very late (in the last 30 days before death), 25.8% (*N* = 785) were referred late (31 to 90 days before death) and 21.4% (*N* = 652) patients were referred early (more than 3 months before death).

**Fig. 2:**
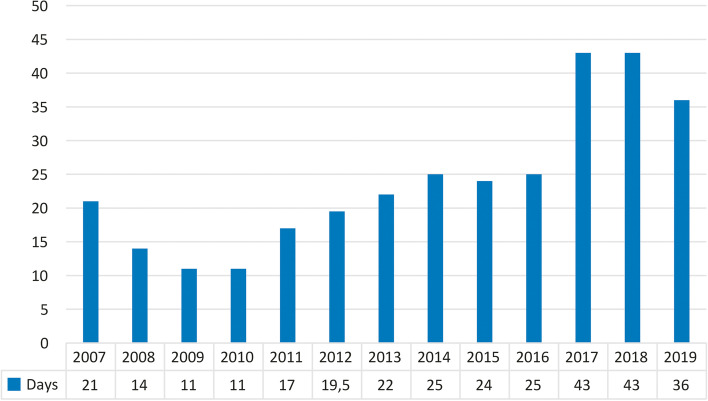
Annual trends in median referral-to-death intervals

No difference was observed between males and females in referral-to-death intervals (*p* = 0.16). There was also no significant difference in median referral-to-death interval among predefined age groups (15–45 years—25.5 days, 41–65 years—23 days, 66–75 years—25 days, 76–85 years—27 days and 86–100 years—33.5 days, *p* = 0.493). There was no difference in median referral-to-death interval in patients aged below and above 75 years either (median 24 and 27 days, respectively, *p* = 0.165). Therefore, age did not correlate with the duration of referral time (ρ = 0.03, *p* = 0.08). The annual trends of median referral-to-death intervals are given in Fig. 2.

**Fig. 3:**
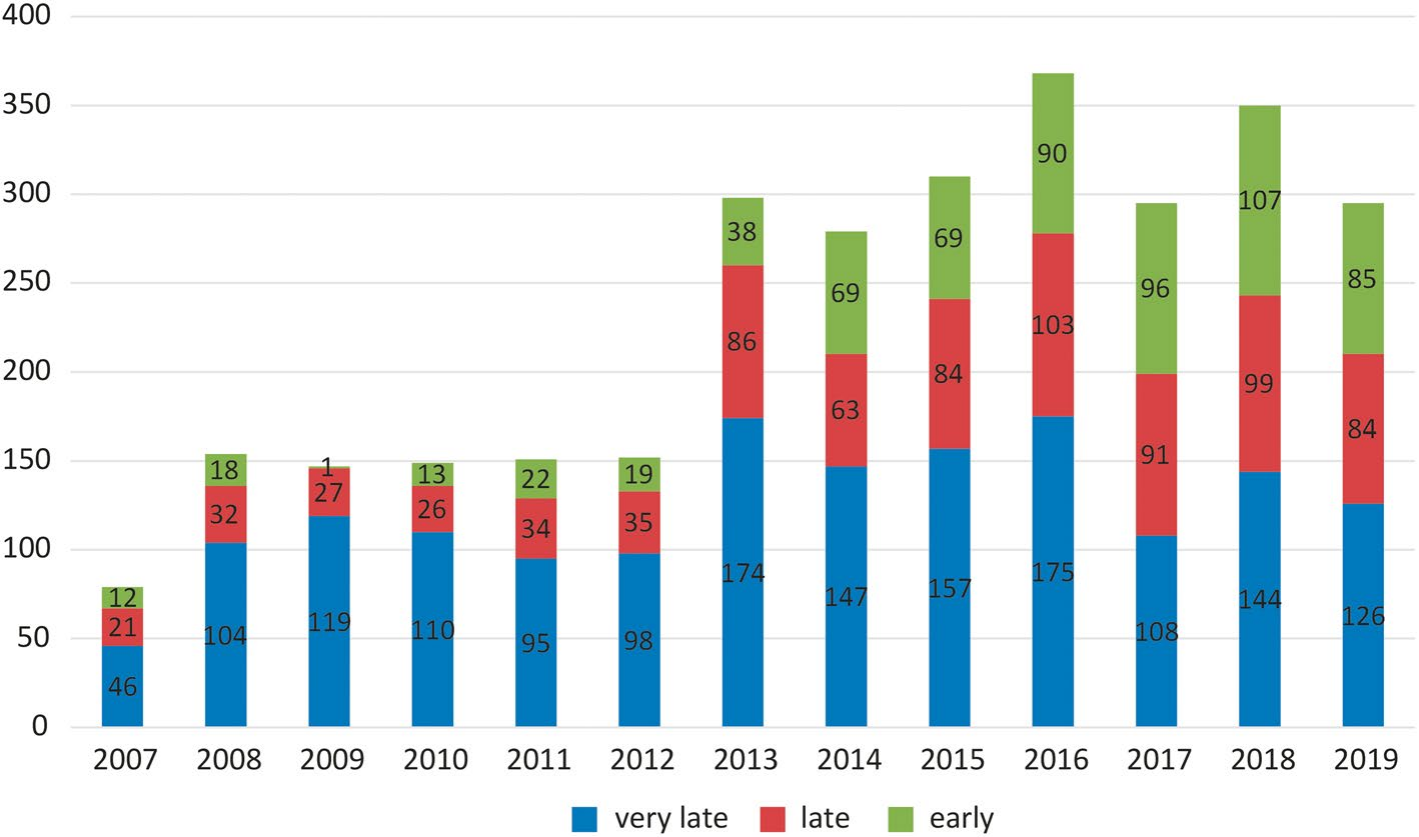
Trends in annual referral-to-death intervals, divided into three time groups (early, late and very late)

Trends in annual referral-to-death intervals with regard to timeliness of referrals (early, late, very late) are given in Fig. 3.

There was a strong correlation between the year of referral to palliative care with the duration of referral time (ρ = 0.19, *p* <  < 0.001). When we divided the observation period into two intervals – the first before opening of the outpatient clinic in 2013 and the second after that, the correlation was significant (ρ = 0.19, *p* < 0.001).

**Fig. 4:**
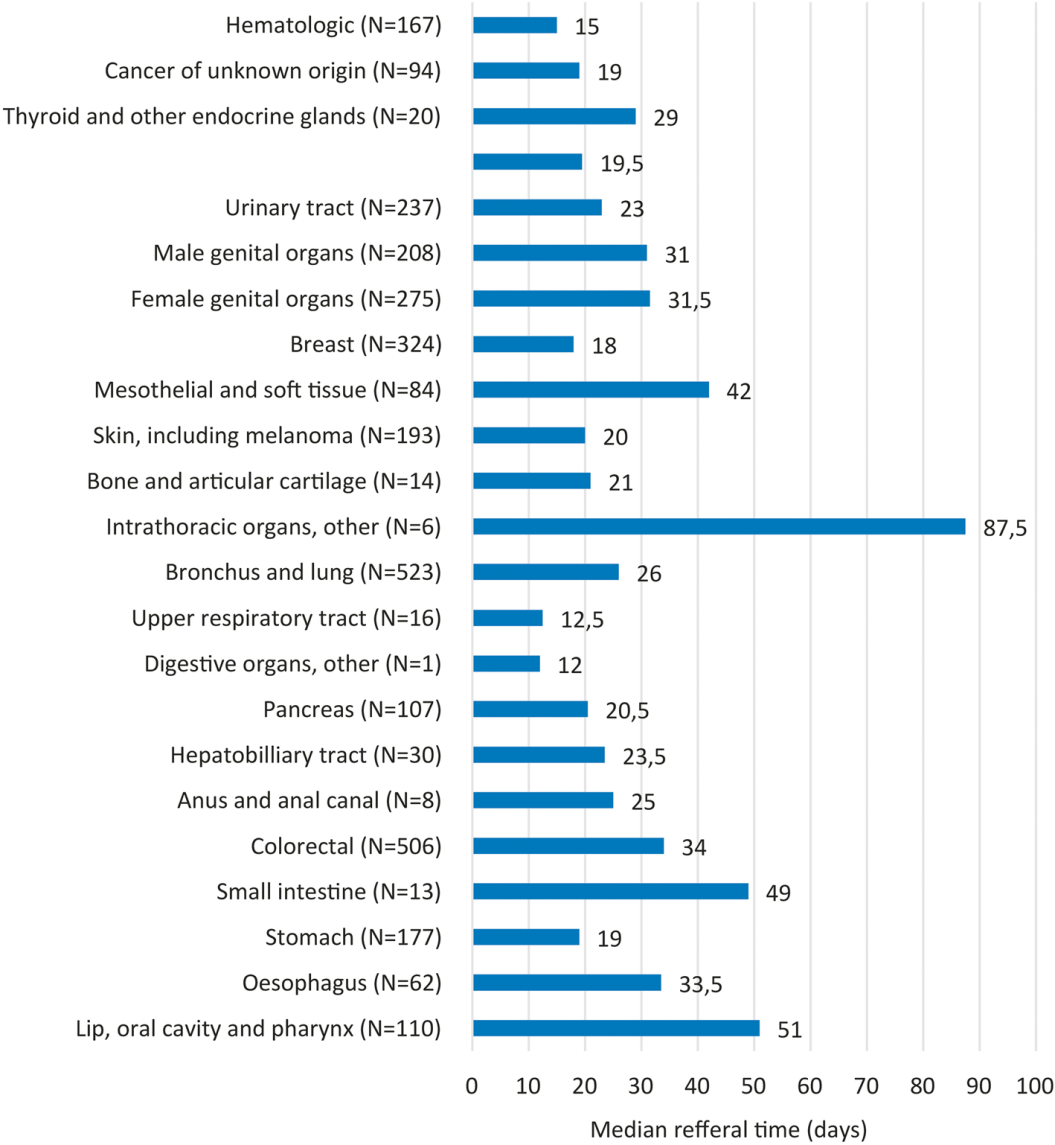
Median referral-to-death interval for all primary cancer sites

Median referral-to-death interval for all primary cancer sites are given in Fig. 4. The primary site actually correlated with the referral-to-death interval (*p* <  < 0.001), which can be clearly seen in Fig. 4.

When comparing groups of malignancies with a higher incidence, we observed a statistically significant difference in referral-to-death intervals between patients with lung and breast cancer (26 and 18 days, respectively, *p* = 0.049) and patients with colorectal and breast cancer (34 and 18 days, respectively, *p* = 0.001), and a borderline difference between patients with lung and colorectal cancer (26 and 34 days, respectively, *p* = 0.056).

## Discussion

This retrospective assessment analyzes real life specialized palliative care in more than 3000 patients over 13 years from 2007 to 2019.

In a similar study performed in Leeds, UK, analysing 3900 cancer patients (median age 75 years), among patients with non-cancer diagnoses, patients over 75 had limited access to palliative care compared to younger patients [9]. A Brazilian report also demonstrated that younger patients received palliative consultations more frequently than elderly patients [8]. In our assessment (median age 67), there was no difference in median referral-to-death interval between patients aged below 75 and those aged above 75 years (*p* = 0.165) and we found no correlation of age with the duration of referral time. We consider this an encouraging statistic regarding our practice with elderly patients. Equal accessibility regardless of patient age should be the goal of every oncological center practicing palliative care.

Another important finding from our analysis was an increasing trend in early SPC refferals: in 2007, as few as 15% of patients were referred more than 3 months prior to death, while in 2017, 2018 and 2019, early referrals comprised 32, 31 and 29% of all referred patients, respectively. The increasing percentage of patients referred early is an important development in palliative care centers for all the aforementioned reasons.

Bennett’s study [9], covering a period between April 2012 and March 2014, reported a median referral-to-death interval of 37 days, which appeared better compared to our results (25 days prior to death). However, from the time of establishment of our SPC inpatient service in 2007, the referral-to-death intervals at our institution increased simultaneously with the upgrade of our SPC services to include outpatient and inpatient consultation services in 2013. During the last three years, we have observed that most of the referrals exceeded 30 days before death, aligning us with MD Anderson’s study from 2012 [10] (1.4 months) and with Bennett’s numbers reported in 2016 (37 days) [9] and exceeding the recently published review of 169 studies worldwide with a median interval-to-death of 18.9 days, specifically 15 days for patients with cancer [12]. On the other hand, the Brazilian group actually reported a worsening in referral time during their 5 year observation period [8], which only underlines our achievement of elongation of the referral time and increase in the percentage of patients referred early.

The majority of patients in our cohort had lung cancer, colorectal cancer and breast cancer, reflecting the higher incidences of those diseases. Our lung cancer specialists deal with other intrathoracic malignancies as well as with some of the upper airway tract tumour malignancies, signifying that these early referrals are consistent with their awareness of the integration of palliative care into oncologic care, which has been persuasively influenced by Temel’s article in 2010 [3]. Interestingly, in Bennett’s study, breast cancer patients were admitted to palliative care units very early [9], and the same was observed in Hui’s study [10], whereas in our assessment, breast cancer patients were among the patients having the latest referrals to SPC (18 days before death). We partially attribute this fact to the wide availability of drugs for the treatment of breast cancer in Slovenia*,* which means that oncologists try all treatment options possible prior to palliative care referral. In addition, due to the lack of properly trained physicians in Slovenia and the limited capacities of the SPC team, the majority of oncologists practice palliative care for their patients by themselves and consult the SPC team only for particularly difficult cases.

Patients with hematological malignancies are known to be referred late and infrequently [10], yet Bennett’s median referral time [9] was still longer compared to the one reported for our hematological patients (26 vs. 15 days, respectively). An Australian group reported a median time of 23 days from the first contact with the palliative team to death for patients with non-Hodgkin’s lymphoma [13]. In our assessment, the majority of hematological patients had aggressive lymphomas, which are generally treated aggressively and intensively throughout the entire course of their disease, while patients with low-grade lymphomas represented only a minority of patients. The analysis of Hui and his colleagues showed that solid tumour oncologists perceived themselves as more comfortable with offering supportive and palliative care to their patients than hematologic oncologists [14], which is also worth considering. Our hematologic oncologists usually employed SPC consultations near the end of life, as their patients were rarely eligible for the outpatient clinic for the aforementioned reasons.

There are some limitations of our study – this is a single institution analysis and there are difficulties of direct comparison with other centres as the characteristics and organization of palliative care services are different in almost every country in the world. The main advantages of this study are the high number of cancer patients included, the different cancer diagnoses included and represented in high numbers, the broad time interval of evaluation demonstrating development throughout these years, and the correlations performed to show the comparable availability of SPC services for our patients.

## Conclusion

The integration of early palliative care into oncological care is an element of almost all cancer guidelines, though it is in practice rarely achieved. This real-life data analysis of a large number of patients revealed no correlations of age or sex to referral-to-death interval, but did demonstrate the expected differences regarding referrals of patients with different primary cancer sites. Our study showed that equal accessibility is possible even during the years of development in palliative care services, whether for national centres or regional institutions establishing their palliative network.

## Data Availability

The datasets used and/or analyzed during the current study are available from the corresponding author upon reasonable request.

## Abbreviations

IOL: Institute of Oncology Ljubljana
SPC: Specialized palliative care
APCD: Acute Palliative Care department
